# A Case of Polymetatarsia Without Polydactyly

**DOI:** 10.7759/cureus.9730

**Published:** 2020-08-14

**Authors:** Steven R Edwards

**Affiliations:** 1 Surgery, Australasian College of Podiatric Surgeons, Melbourne, AUS; 2 Podiatry, La Trobe University, Bundoora, AUS

**Keywords:** metatarsal, anatomic variant, foot surgery techniques, polymetatarsia, polydactyly

## Abstract

Polymetatarsia is an atavistic anomaly characterised by one or more additional metatarsals. Usually found with a supernumerary digit (polydactyly), polymetatarsia without polydactyly is a rare variant. We report a case of a 34-year-old male with polymetatarsia within the first intermetatarsal spaces of both feet without polydactyly. Clinically, moderate dorsal spur formation was visible, and compressive pain from ankylosed additional metatarsals within the first intermetatarsal spaces was exhibited. Treatment involved resection of his additional metatarsals with concomitant correction of his hallux valgus deformities and bilateral second brachymetatarsia. He reported a reduction in pressure and pain that was maintained until his discharge appointment at six weeks postoperatively. Resection of additional metatarsals may provide effective pain relief in symptomatic patients.

## Introduction

Polymetatarsia is an atavistic anomaly characterised by one or more additional metatarsal bones that is usually accompanied by an extra digit. Polymetatarsia without a supernumerary digit is a rare variant, and only a few cases have been reported [1-4]. Herein, a case is reported of a 34-year-old male with polymetatarsia within the first intermetatarsal spaces of both feet and his subsequent treatment.

## Case presentation

**Figure 1:**
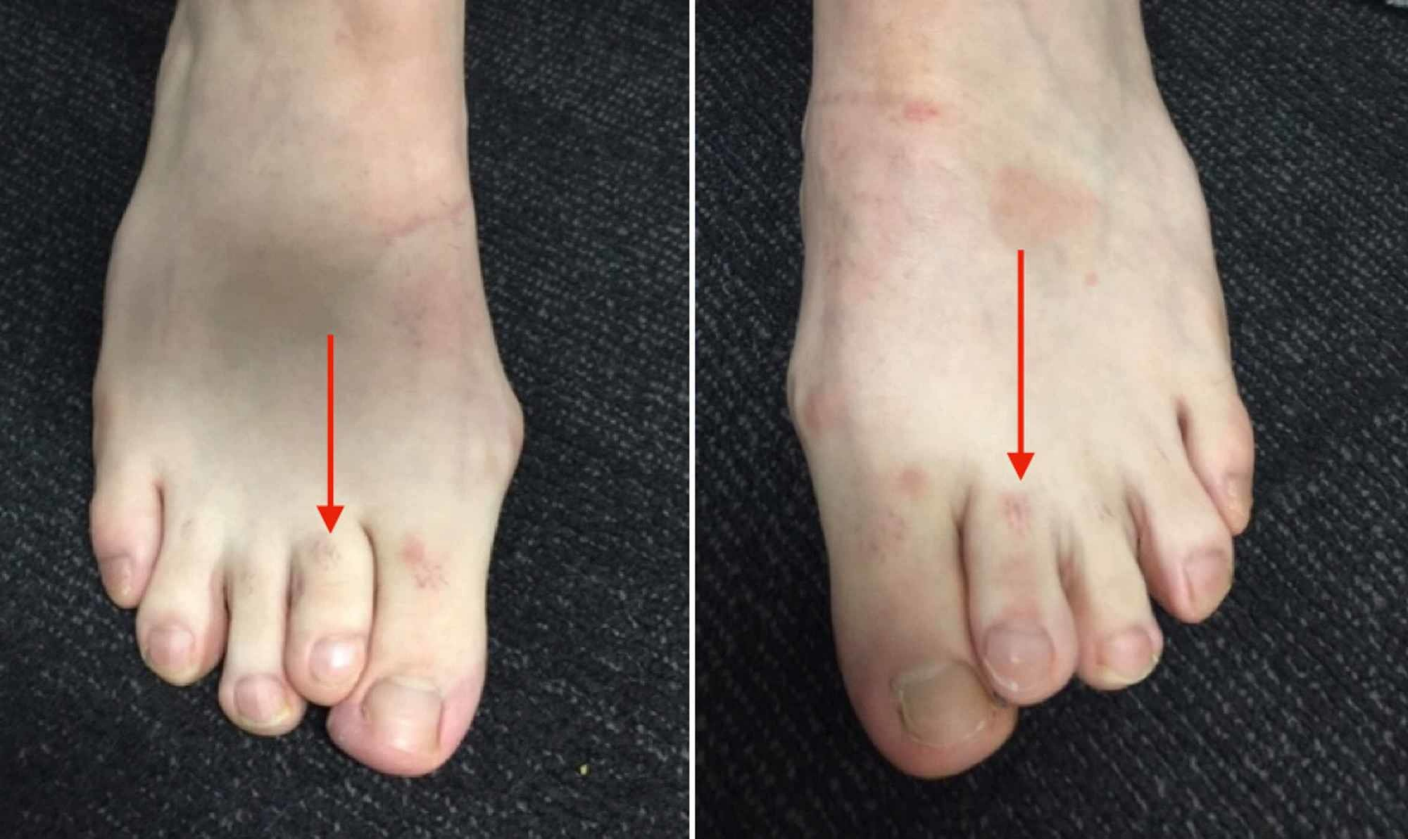
The dorsal bulges are difficult to visualise clinically but can be palpated. Both second metatarsals exhibit brachymetatarsia (red arrows).

A 34-year-old male patient was referred with pain and pressure within both first intermetatarsal spaces and for the correction of the brachymetatarsia affecting both second rays. His right second digit was one centimetre shorter than the left foot (Figure 1).

**Figure 2:**
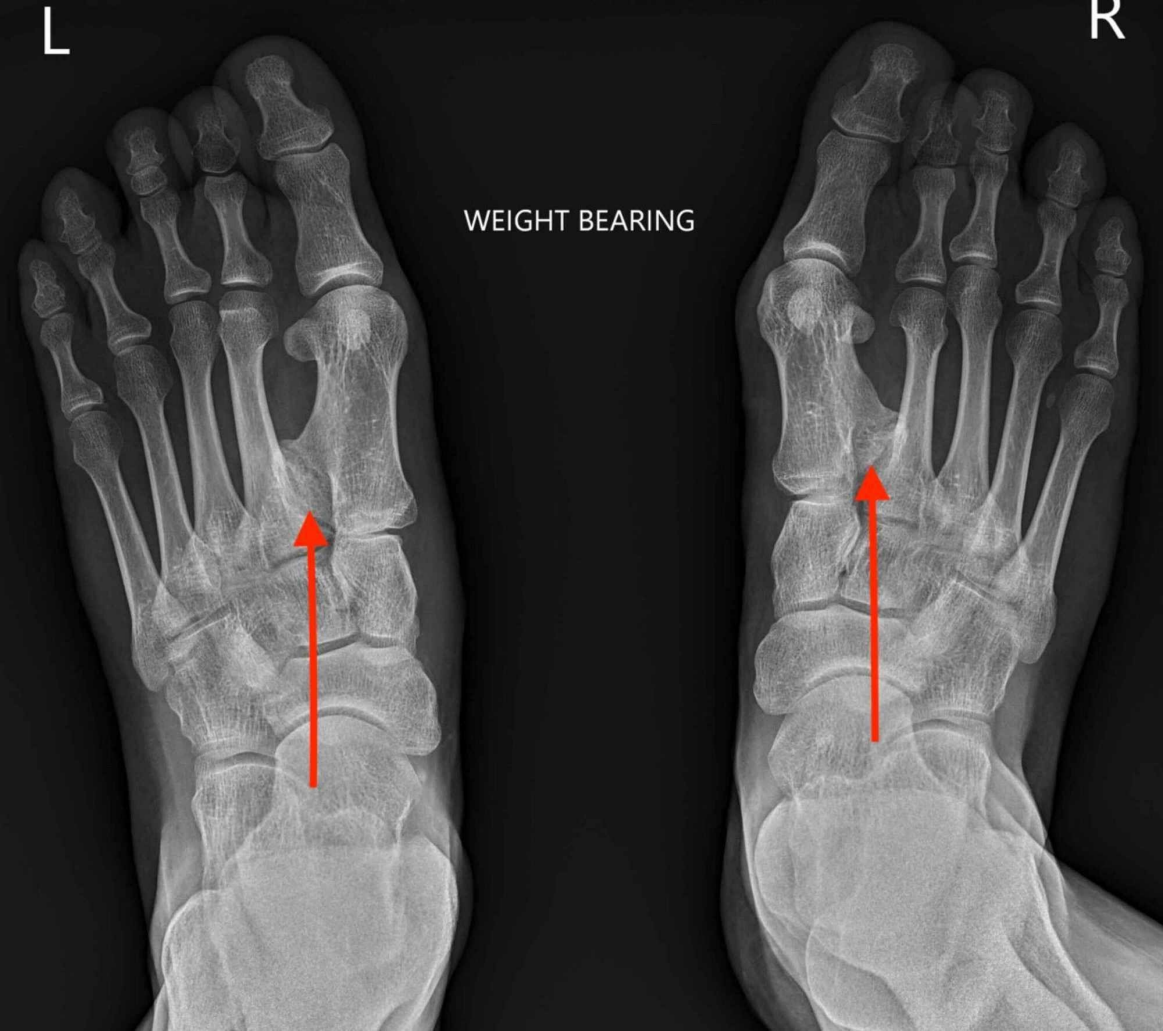
Ankylosed polymetatarsia seen within the first intermetatarsal spaces of both feet (red arrows). Note the additional lack of lesser toe distal interphalangeal joints.

**Figure 3:**
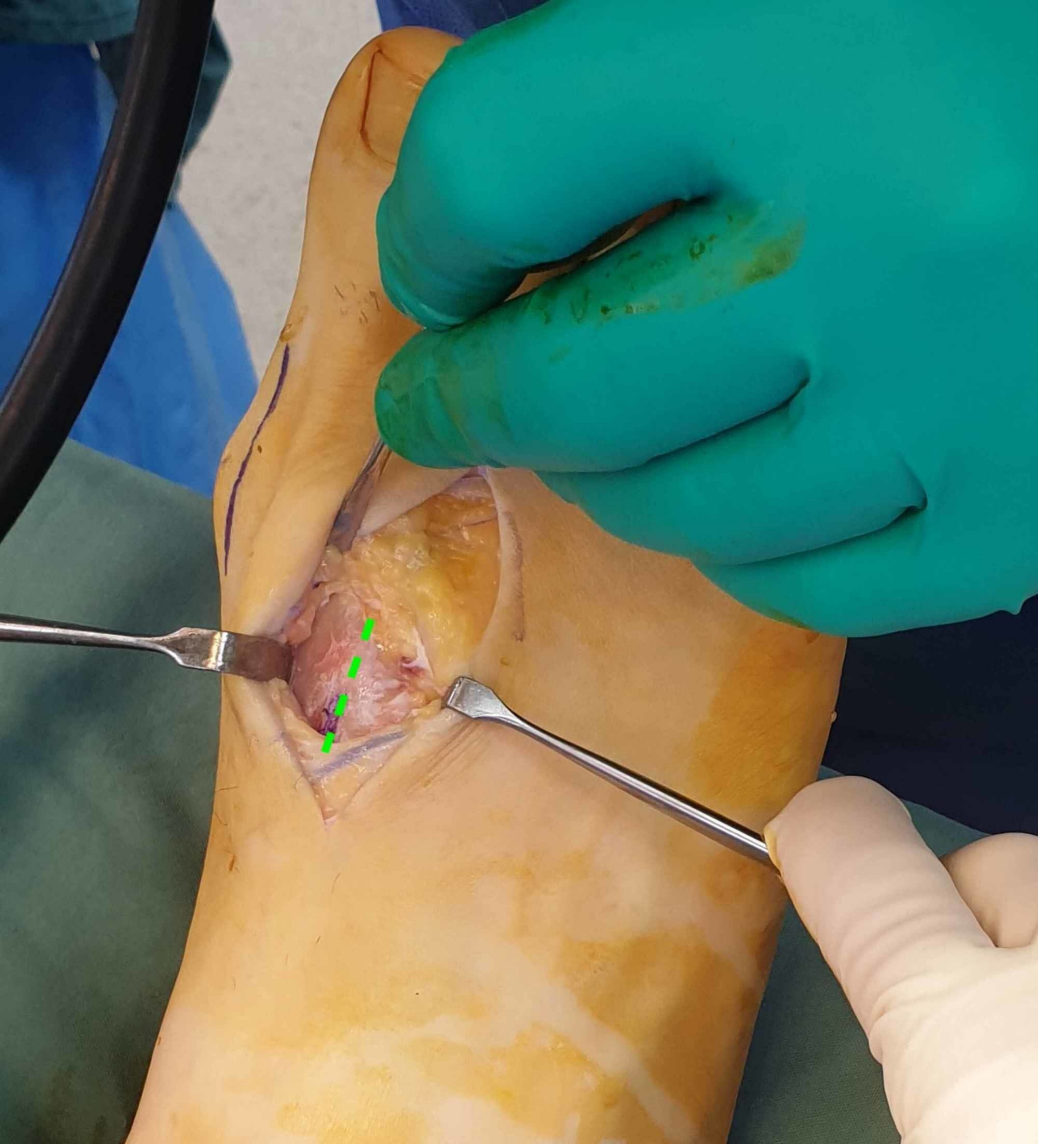
The cleavage point between the variant and the first metatarsal is identified (green line).

Radiographs (Figure 2) showed polymetatarsia within the first intermetatarsal spaces, and immediate preoperative fluoroscopy (Figure 3) illustrating the extent of the additional metatarsals.

 

**Figure 4:**
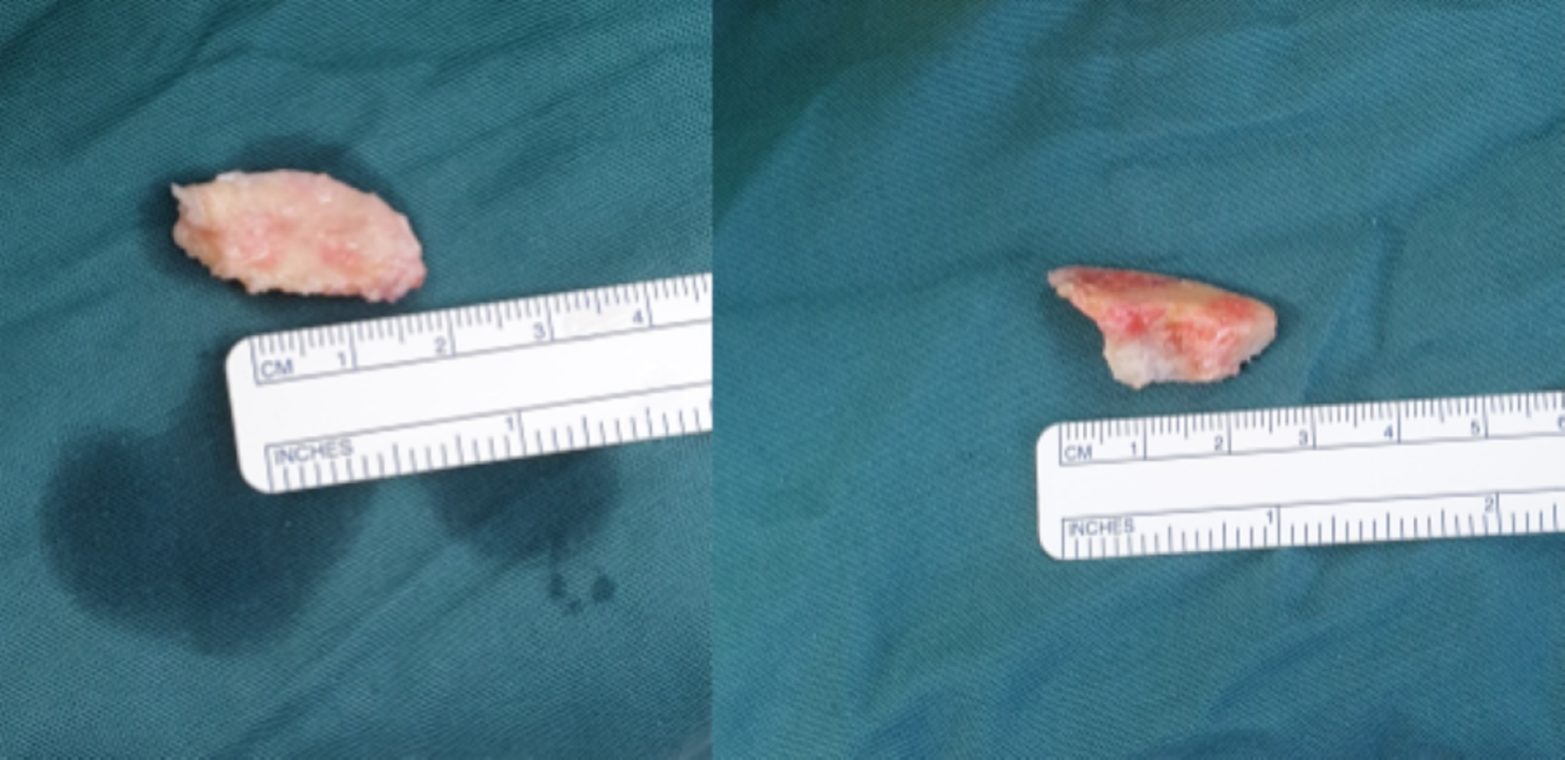
The variant within the right first intermetatarsal space is resected in two fragments.

**Figure 5:**
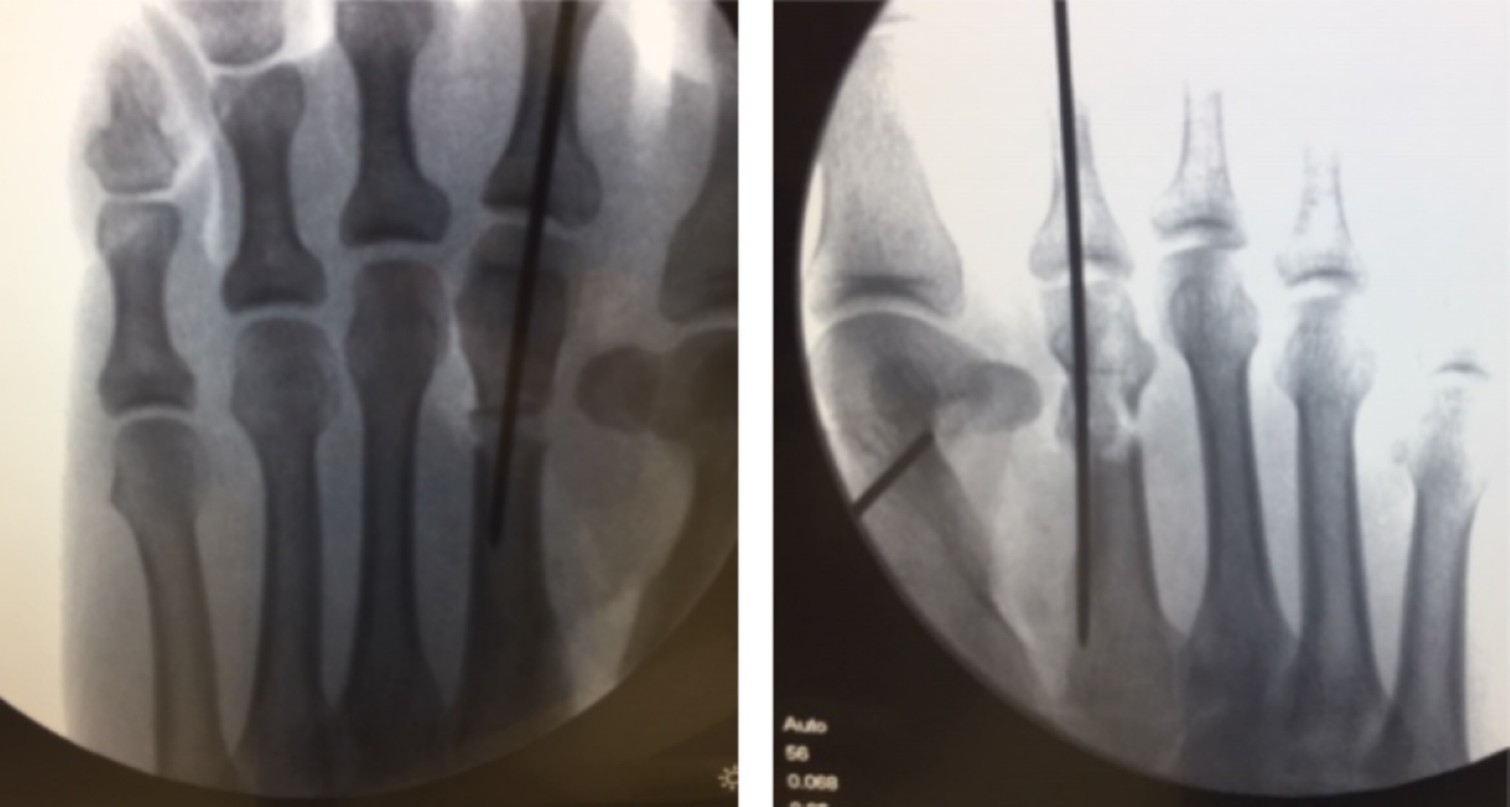
Immediate postoperative fluoroscopy showing decompression of the first metatarsal interspaces.

As conservative treatment had not reduced his symptoms, surgery was performed to relieve the pain and pressure. The variants were exposed via dorsal curvilinear incisions along the length of the first intermetatarsal spaces to allow for resection and lengthening of the shortened second metatarsals (Figure 3). His hallux valgus deformities were corrected concomitantly. The variants were resected (Figure 4), and the second metatarsals lengthened using bone graft from the fragments. Immediate postoperative fluoroscopy (Figure 5) showed decompression of the first intermetatarsal spaces and re-establishment of the metatarsal parabola. 

The patient reported immediate reductions in pressure following resection. This was maintained at six weeks postoperatively until his discharge. 

## Discussion

The differentiation of the human limbs occurs from proximal to distal and is divided into four stages: patterning of skeletal elements, formation of the individual condensations, elongation/segmentation of the condensations, and growth/differentiation. Molecular studies of isolated forms of brachydactyly have informed researchers on the role of certain genes in normal human skeletogenesis and limb formation [5-7].

**Figure 6:**
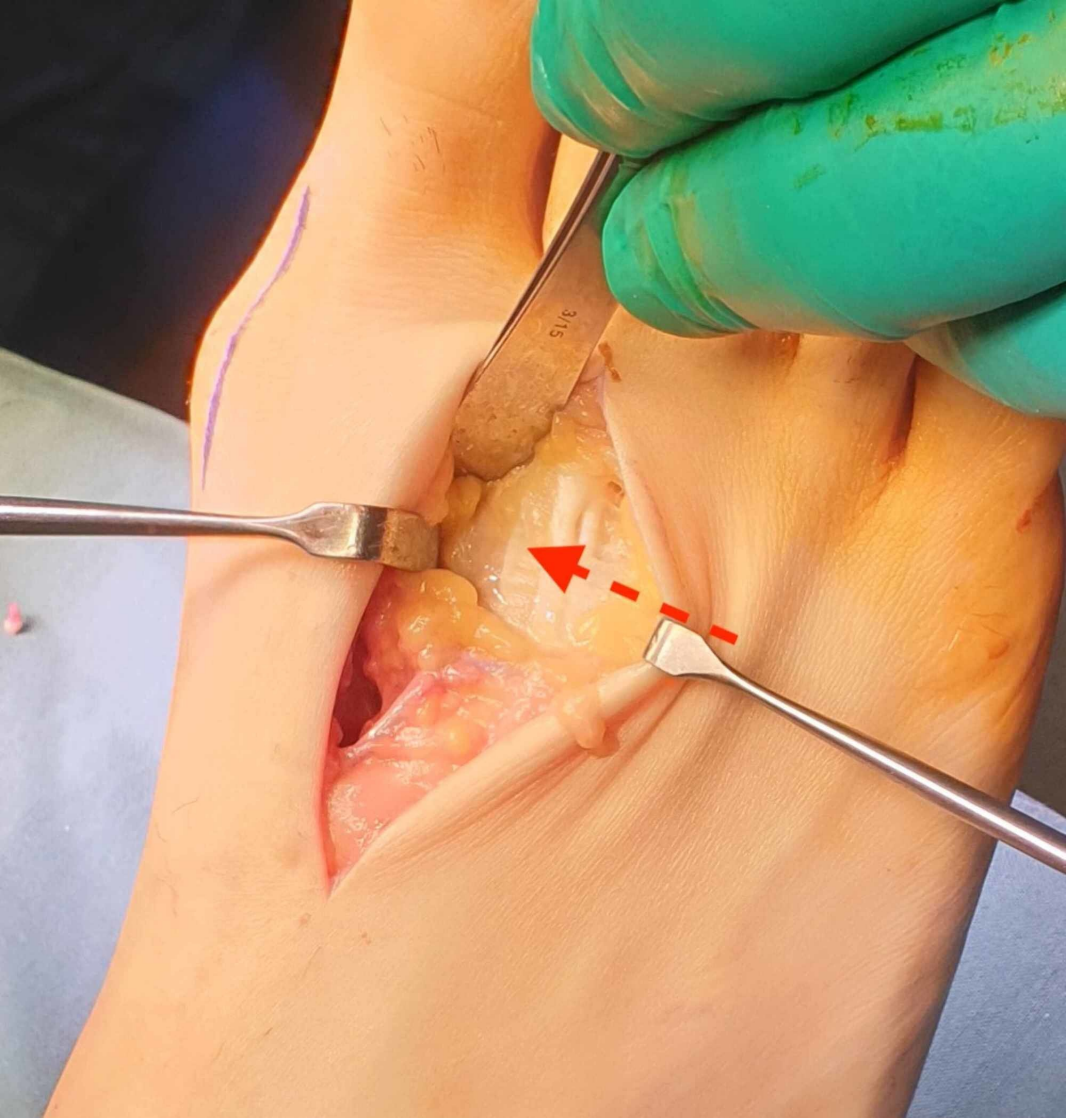
An extensor digitorum accessorius accessory tendon is also observed (red arrow).

Polymetatarsia forms from embryological overinduction of the digital rays [5]. In our case, other atavistic variants were also exhibited. The patient lacked distal interphalangeal joints in all lesser digits and an extensor digitorum accessorius tendon was present (Figure 6). 

The diagnosis of polymetatarsia may be troublesome, and it is often confused with an os intermetatarseum ossicle. A feature differentiating the two is the presence of a growth plate. Usually, polymetatarsia occurs within the fourth intermetatarsal space, but may in theory occur within any [2]. 

We speculated whether the polymetatarsia impacted the development of both second metatarsals, as both were short and the right was one centimetre shorter than the third metatarsal. This seems probable; however, it remains unconfirmed.

## Conclusions

A case of bilateral polymetatarsia without polydactyly in a 34-year-old male is reported. Resection of the variants and re-establishment of his metatarsal parabola ameliorated his pain. If symptomatic, resection of the supernumerary metatarsals may be effective for reducing pain and deformity.
